# Identification of Protein Subcellular Localization With Network and Functional Embeddings

**DOI:** 10.3389/fgene.2020.626500

**Published:** 2021-01-20

**Authors:** Xiaoyong Pan, Hao Li, Tao Zeng, Zhandong Li, Lei Chen, Tao Huang, Yu-Dong Cai

**Affiliations:** ^1^School of Life Sciences, Shanghai University, Shanghai, China; ^2^Key Laboratory of System Control and Information Processing, Institute of Image Processing and Pattern Recognition, Shanghai Jiao Tong University, Ministry of Education of China, Shanghai, China; ^3^College of Food Engineering, Jilin Engineering Normal University, Changchun, China; ^4^Bio-Med Big Data Center, CAS Key Laboratory of Computational Biology, CAS-MPG Partner Institute for Computational Biology, Shanghai Institute of Nutrition and Health, Chinese Academy of Sciences, Shanghai, China; ^5^College of Information Engineering, Shanghai Maritime University, Shanghai, China; ^6^Key Laboratory of Tissue Microenvironment and Tumor, Shanghai Institute of Nutrition and Health, Chinese Academy of Sciences, Shanghai, China

**Keywords:** protein subcellular localization, network embedding, functional embedding, gene ontology, KEGG pathway

## Abstract

The functions of proteins are mainly determined by their subcellular localizations in cells. Currently, many computational methods for predicting the subcellular localization of proteins have been proposed. However, these methods require further improvement, especially when used in protein representations. In this study, we present an embedding-based method for predicting the subcellular localization of proteins. We first learn the functional embeddings of KEGG/GO terms, which are further used in representing proteins. Then, we characterize the network embeddings of proteins on a protein–protein network. The functional and network embeddings are combined as novel representations of protein locations for the construction of the final classification model. In our collected benchmark dataset with 4,861 proteins from 16 locations, the best model shows a Matthews correlation coefficient of 0.872 and is thus superior to multiple conventional methods.

## Introduction

The functions of proteins are closely related to their subcellular locations in cells. In studying proteins, determining their locations in cells is usually the first step, and these locations are used as guides for designing drugs. Thus, many experimental methods for identifying protein locations have been developed, such as *in situ* hybridization. Through these methods, a large number of proteins have been verified and recorded in biological databases, such as the Swiss-Prot database. In addition, these data serve as benchmark datasets for developing machine learning methods and useful in the computational identification and investigation of protein locations.

Many computational methods based on machine learning for predicting protein subcellular locations have been proposed. For example, Chou and Cai (2002) proposed a support vector machine-based method for predicting protein locations with the use of functional domain data. LocTree2 (Goldberg et al., 2012) presents a hierarchical model for classifying 18 protein locations. To further improve prediction effectiveness, LocTree3 incorporates homology information into the models (Goldberg et al., 2014). Hum-mPloc 3.0 trains an ensemble classifier by integrating sequence and gene ontology information (Zhou et al., 2017). Recently, deep learning has achieved remarkable results in computational biology, particularly in identifying protein subcellular locations. In classification tasks, deep learning automatically learns high-level features rather than hand-designing features. For example, DeepLoc (Almagro Armenteros et al., 2017) presents a recurrent neural network with attention mechanism for the identification of protein locations, using sequences alone. rnnloc (Pan et al., 2020a) combines network embeddings and one-hot encoded functional data to predict protein locations with the use of a recurrent neural network. In Hum-mPloc 3.0 and rnnloc, functional data demonstrate strong discriminating power for different subcellular locations. However, both methods encode functional data into a high-dimensional one-hot encoded vector, which may cause feature disaster, especially when the number of training samples is smaller than the number of features.

For the above issues, embedding-based methods can be applied to the transfer of high-dimensional one-hot encoding into distributed vectors for sequential and network data. Given that interacting proteins generally share similar locations, node2vec (Grover and Leskovec, 2016) can be used in learning network embeddings for individual proteins from a protein–protein network, which help better represent the protein interaction information into feature vectors.

In this study, we present an embedding-based method for predicting protein locations. It learns network embeddings from a protein–protein network and functional embeddings of GO/KEGG terms. Then, these learned embeddings are used to represent proteins and further selected using feature selection methods. Finally, an optimal feature subset and classifier are obtained for the classification of protein subcellular localization, and the optimal classifier is superior to multiple conventional methods.

## Materials and Methods

**Figure 1:**
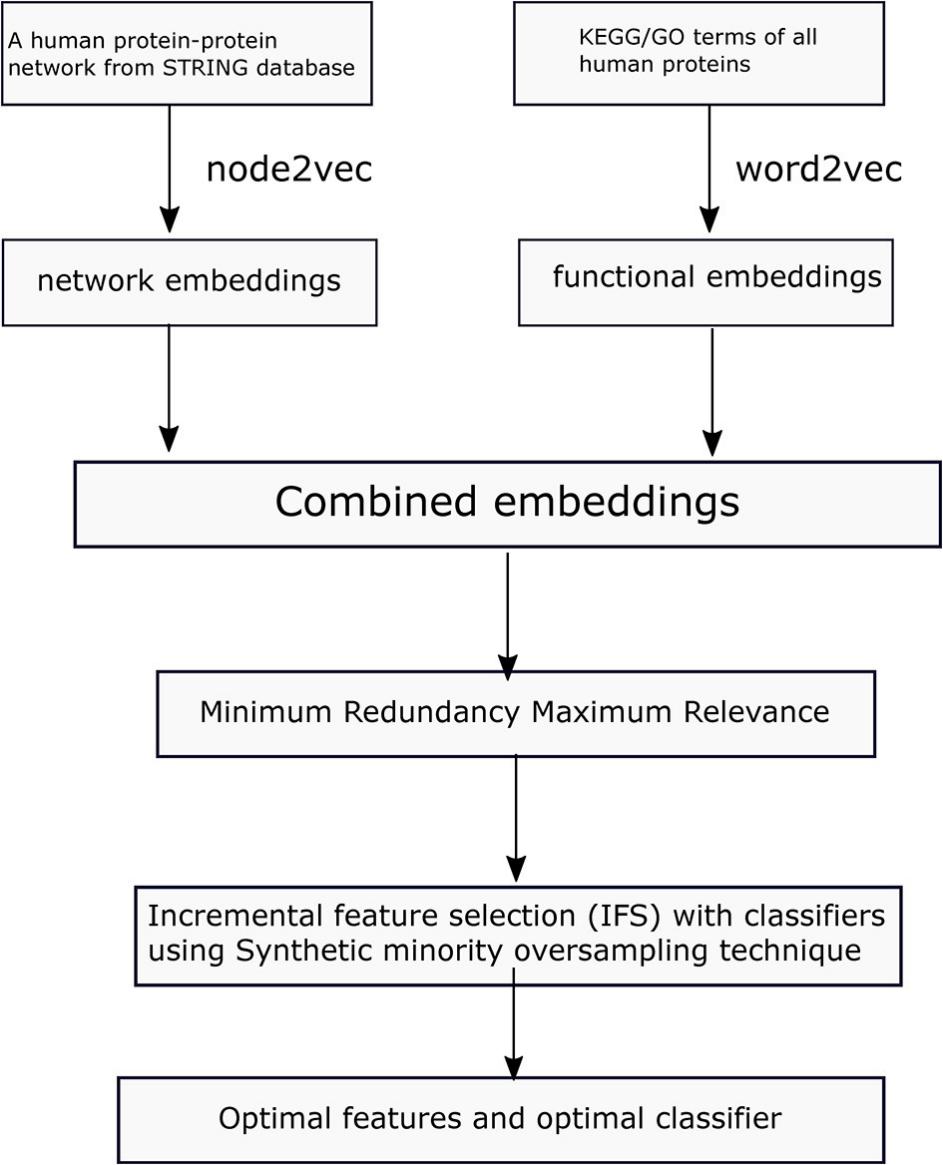
Flowchart of the proposed method in this study.

In this study, we first collect a benchmark dataset for protein localization. Then we learn network embeddings from a protein–protein network, using node2vec and functional embeddings from KEGG/GO functional data and word2vec. Then, the learned embeddings are used to represent each protein. To obtain refined combined embeddings, we use two-step feature selection methods in determining the optimal features and classifiers in predicting protein locations. The whole process is illustrated in Figure 1.

### Datasets

The original 5,960 protein sequences are retrieved from a previous study (Li et al., 2014), which are extracted from Swiss-Prot (http://cn.expasy.org/, release 54.0). The protein sequences do not include proteins with <50 amino acids or more than 5,000 amino acids and unknown amino acids. The included proteins are processed through CD-HIT (Li and Godzik, 2006). Sequence similarity between each pair of proteins is <0.7. Given that we extract features from gene ontology (GO) terms and KEGG pathways of proteins through natural language processing methods, we exclude proteins without GO terms and KEGG pathways, finally obtaining a total of 4,861 proteins. The proteins are classified into 16 categories according to their subcellular locations. The number of proteins from each location is listed in Table 1.

**Table 1:** Number of proteins in each category.

**Category**	**Number of proteins**
Biological membrane	1,483
Cell periphery	33
Cytoplasm	488
Cytoplasmic vesicle	69
Endoplasmic reticulum	188
Endosome	25
Extracellular space or cell surface	636
Flagellum or cilium	3
Golgi apparatus	95
Microtubule cytoskeleton	48
Mitochondrion	326
Nuclear periphery	31
Nucleolus	108
Nucleus	1,229
Peroxisome	45
Vacuole	54

### Protein Representation

#### One-Hot Encoded Representation Based on GO Term and KEGG Pathway

The GO terms and KEGG pathways are the essential properties of proteins. A representation containing this information is an excellent scheme for encoding each protein.

A protein *p* can be encoded into a binary vector, *V*_*GO*_(*p*), based on its GO terms, which is formulated by

(1) $$\begin{array}{l} V_{GO}\left(P\right)={\left[g_{1},g_{2},\cdots \;,g_{m}\right]}^{T}, \end{array}$$

where *m* represents the number of GO terms and *g*_*i*_ is defined as follows:

(2) $$\begin{array}{l} g_{i}=\{\begin{array}{cc} 1 & if\;p\;is\;annotated\;by\;the\;i-th\;GO\;term\;\\ 0 & Otherwise \end{array} \end{array}$$

In this study, 22,729 GO terms are included, which induced a 22,729-D vector for each protein.

Moreover, with its KEGG pathways, it can also be encoded into a vector, *V*_*KEGG*_(*p*), with the formula

(3) $$\begin{array}{l} V_{GO}\left(P\right)={\left[k_{1},k_{2},\cdots \;,k_{n}\right]}^{T}, \end{array}$$

where *n* represents the number of KEGG pathways and *k*_*i*_ is defined as follows:

(4) $$\begin{array}{l} k_{i}=\{\begin{array}{cc} 1 & if\;p\;is\;annotated\;by\;the\;i-th\;KEGG\;pathway\;\\ 0 & Otherwise \end{array} \end{array}$$

Here, 328 KEGG pathways are used, inducing a 328-D vector for each protein.

Two vectors based on GO terms and KEGG pathways are concatenated to a final vector. Thus, each protein is represented by a 23,057-D vector. We use Boruta feature selection (Kursa and Rudnicki, 2010) to reduce the computational burden and retain relevant features.

#### Functional Embeddings Based on GO Term and KEGG Pathway

Given that the one-hot encoded representation of GO (Ashburner et al., 2000) and KEGG (Ogata et al., 1999) terms is highly dimensional, the method by which they are mapped into low-dimensional embeddings is extremely important. GO/KEGG terms co-occur in a protein frequently and may thus be similar in distance, although distances among GO/KEGG terms vary. Thus, we apply word2vec (Mikolov et al., 2013) to learn an in-depth representation of GO/KEGG terms, representing each GO/KEGG term with a vector containing continuous values.

We first collect whole human proteins with GO/KEGG terms. Each GO or KEGG term is a word, and each protein is a sentence. The set of human proteins is a corpus. We run Word2vec program in genism (https://github.com/RaRe-Technologies/gensim) on this corpus to learn the embeddings of each GO/KEGG term.

Each protein contains multiple GO and KEGG terms. After obtaining the embeddings for each KEGG/GO term, we average the embeddings of KEGG/GO terms within a protein as the functional embeddings of this protein.

#### Network Embeddings From a Protein–Protein Network

In a protein–protein network, each node is a protein, and the edge is whether the two proteins interact or not. We first download a human protein–protein network from STRING (version 9.1) (Szklarczyk et al., 2017), and the network consists of 2,425,314 interaction pairs and 20,770 proteins.

node2vec is designed to learn embeddings from a graph through a flexible sampling approach and maximizes the log probability of nodes, given the learned embeddings:

(5) $$\begin{array}{l} \text{ma}{\text{x}}_{\text{e}}\;\sum_{v\in V}\log P\left(N\left(v|e\left(v\right)\right)\right)\; \end{array}$$

where *v* is the node, *N*(*v*) is the neighborhood of the node *v*, and *e* is the mapping function from nodes to embeddings.

In this study, we use node2vec implemented at https://snap.stanford.edu/node2vec/, and the dimension of the learned embeddings is set at 500. Finally, the network embeddings of each protein are obtained.

### Feature Selection

Instead of directly using combined features from network embeddings and functional embeddings for each protein, we further use minimum redundancy maximum relevance (mRMR) (Peng et al., 2005) to analyze these embedding features, which has wide applications in tackling different biological problems (Wang et al., 2018; Li et al., 2019, 2020; Zhang et al., 2019, 2020; Chen et al., 2020). This method has two criteria to evaluate the importance of features. One is the maximum relevance to class labels and the other is the minimum redundancy to other features. Based on these two criteria, mRMR method generates a feature list, named mRMR feature list. Regardless of relevance and redundancy, mutual information (MI) is adopted in this method to make evaluation. For two variables *x* and *y*, their MI is computed by

(6) $$\begin{array}{l} I(x,y)=\iint p(x,y)\log\frac{p(x,y)}{p(x)p(y)}dxdy, \end{array}$$

where *p*(*x*) and *p*(*x, y*) denote the marginal probabilistic density and joint probabilistic density, respectively. A high MI indicates the strong associations of two variables. The mRMR feature list is produced by adding features one by one. Initially, it is empty. In each round, for each of features not in the list, its relevance to class labels, evaluated by MI value of the feature and class labels, and redundancy to features in the list, assessed by the mean of MI values of the feature and those in the list, are calculated. A feature with maximum difference of relevance to class labels and redundancy to features in the list is picked up and appended to the list. When all features are in the list, the mRMR feature list is complete. Here, we used the mRMR program provided in http://penglab.janelia.org/proj/mRMR/. It is executed with its default parameters.

The mRMR method can only output a feature list. Which features are optimum is still a problem. In view of this, the incremental feature selection (IFS) (Liu and Setiono, 1998) method is employed. This method can extract an optimum feature combination for a given classification algorithm. In detail, from the mRMR feature list yielded by mRMR, IFS generates a series of feature subsets with a step 1, that is, the top feature in the list comprises the first feature subset, the top two features comprise the second feature subset, and so forth. For each feature subset, a classifier is built with a given classification algorithm and samples represented by features in the subset. All constructed classifiers are evaluated by a cross-validation method (Kohavi, 1995). We select the classifier with the best performance and call it as the optimum classifier. The corresponding feature subset is termed as the optimum feature subset and features in this feature subset are denoted as the optimal features.

### Synthetic Minority Oversampling Technique

The number of proteins from different locations varies, resulting in a data imbalance problem. To reduce the impact of data imbalance on classification model construction, we apply synthetic minority oversampling technique (SMOTE) (Chawla et al., 2002) to generate some synthesized samples for minority classes. For each location, except the location with the largest number of proteins, we synthesize new proteins and add them to this location until each location has almost the same number of proteins.

### Classification Algorithm

In this study, we test four classification algorithms to select the best one for our task: decision tree (DT) (Safavian and Landgrebe, 1991), K-nearest neighbors (KNN) (Cover and Hart, 1967), random forest (RF) (Breiman, 2001), and support vector machine (SVM) (Cortes and Vapnik, 1995).

#### K-Nearest Neighbors

KNN is a simple intuitive method for classifying samples. Given a query sample, it calculates the distance between a query sample and training samples. Then. it selects k training samples with the least distance, and the label of the query sample is determined by major voting, which assign a label with the most votes to the query sample.

#### Decision Tree

The DT is an interpretable classifier method, which can automatically learn classification rules from data. It uses a greedy strategy to build a flow-like structure; each internal node is determined by a feature to go to the left or right child node. The leaf node represents the outcome labels. The DT in Scikit-learn implements the CART algorithm with Gini index. It is used in this study.

#### Random Forest

RF (Breiman, 2001; Jia et al., 2020; Liang et al., 2020; Pan et al., 2020b) is a meta predictor with multiple DTs, which are grown from the bootstrap samples consisting of randomly selected features. Given a new sample, RT first uses its multiple trees for the prediction of sample labels, and then majority voting is used in determine the label of the new sample.

#### Support Vector Machine

SVM (Cortes and Vapnik, 1995; Chen et al., 2018a,b; Liu et al., 2020; Zhou et al., 2020) is a supervised classifier based on statistical theory, and it builds a hyperplane with a maximum margin between two classes. It first transforms nonlinear data from a low-dimensional space to a linear high-dimensional space with a kernel trick, then the margin between two classes in the high-dimensional space is maximized for acquisition of SVM parameters. Given a test sample, SVM determines the label according to the side of the hyperplane where it is located.

In this study, we use the Scikit-learn package to implement above four classification algorithms.

### Baseline Methods

#### BLAST

To indicate the utility of the proposed method, we further employ basic local alignment search tool (BLAST) (Altschul et al., 1990) to construct a baseline method and make comparisons. In a given protein sequence, BLAST search the most similar protein sequences, measured with an alignment score, in the training dataset. The method based on BALST directly assigns the class of the most similar protein sequence to a given protein sequence as its predicted class. Such method is evaluated with a Jackknife test.

#### DeepLoc

DeepLoc (Almagro Armenteros et al., 2017) is another deep learning based method for predicting protein locations from sequences. We use DeepLoc downloaded from https://github.com/ThanhTunggggg/DeepLoc with default parameters.

## Results and Discussion

In this section, we first visualize the learned embeddings, using T-SNE, then we evaluate the effectiveness of different classifiers with different input embedding features. Finally, we compare our proposed method with baseline methods.

### Visualization of the Learned Functional and Network Embeddings

**Figure 2:**
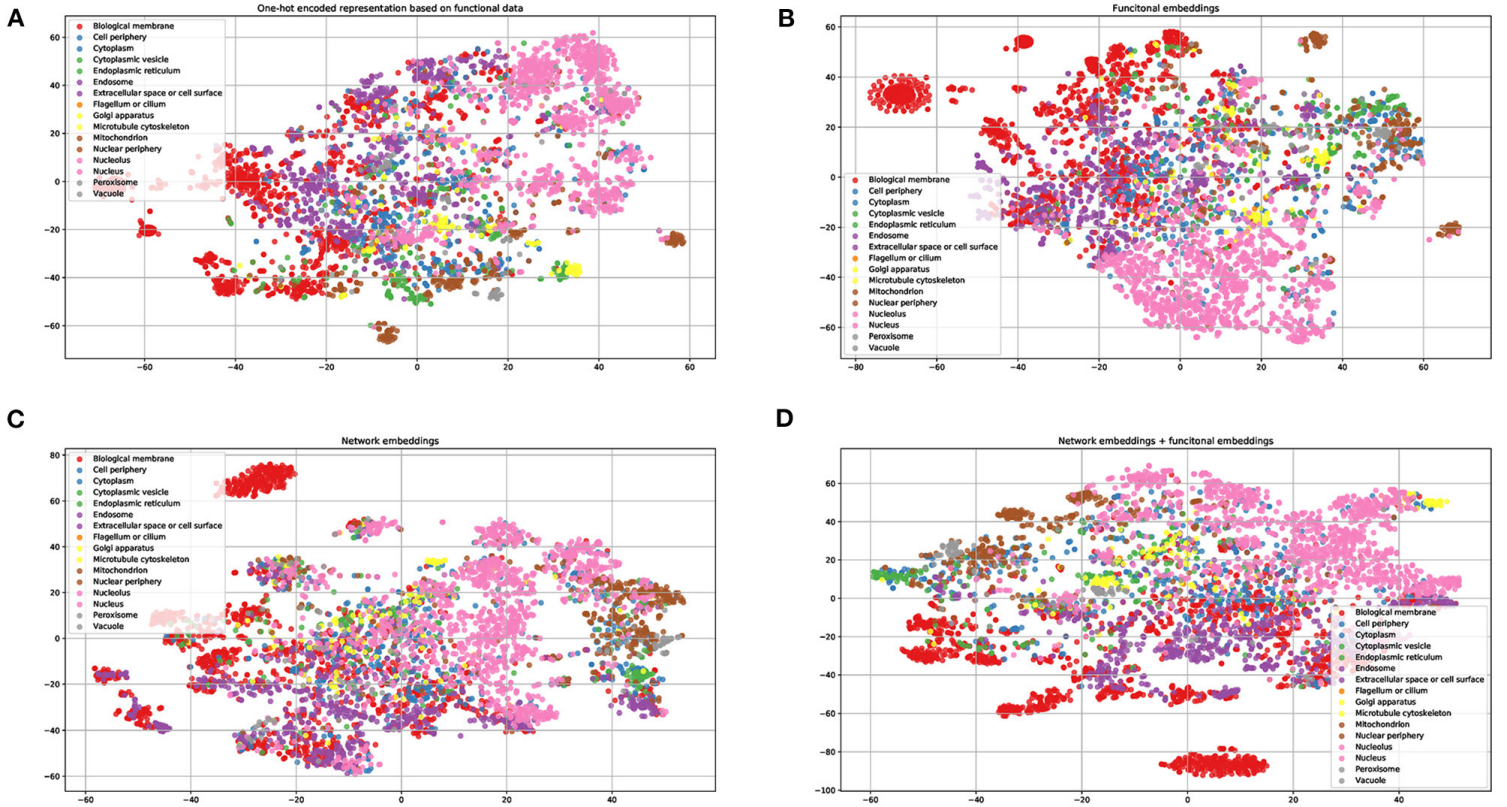
Visualization of one-hot encoded functional features and the learned embeddings. **(A)** One-hot encode representation of functional data KEGG/GO terms; **(B)** the learned network embeddings from a protein-protein network; **(C)** the learned embeddings of functional data using word2vec; **(D)** the combined network and functional embeddings.

To demonstrate the power of the learned embeddings, we visualize these embeddings, one-hot encoded features, and the combined network and functional embeddings, respectively. As shown in Figure 2, the embeddings can distinguish proteins from different locations to some extent. The learned functional embeddings (Figure 2B) shows higher discriminate power on some locations (e.g., for discriminating biological membrane) than the one-hot encoded representation based on functional data (Figure 2A). As shown in Figure 2C, the network embeddings have some discriminate power for some locations, for example, endosomes, which cannot be easily separated by functional embeddings. Also, the combined embeddings (Figure 2D) of functional and network embeddings have strong discriminating power. Intuitively, the four types of embeddings have similar discriminating power on the whole.

### Effectiveness of Different Classifiers With Different Input Embedding Features

**Figure 3:**
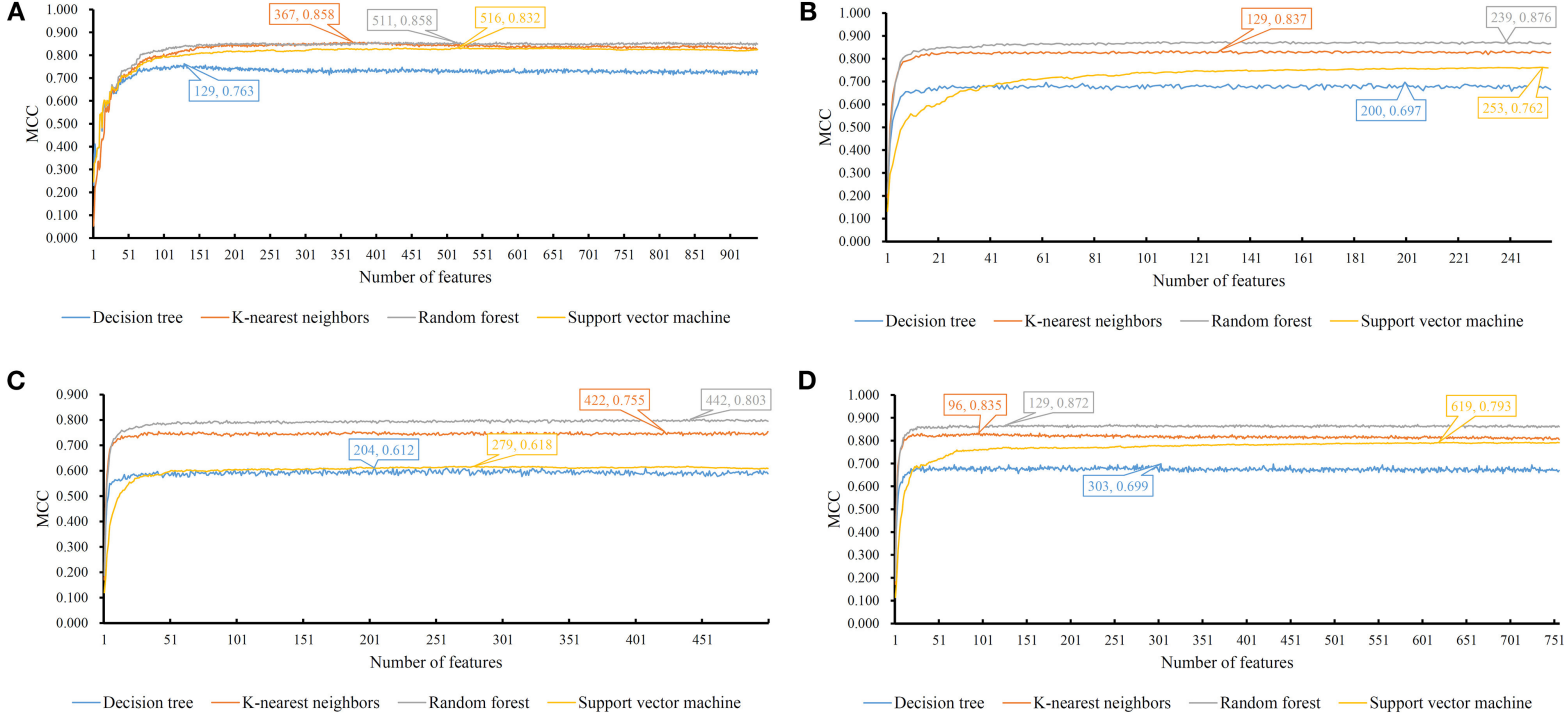
MCC changes with the number of features for IFS with different classification algorithms. **(A)** one-hot encoded representation derived from KEGG/GO terms; **(B)** functional embeddings from KEGG/GO terms; **(C)** network embeddings from a protein-protein network; **(D)** the combined functional and network embeddings.

We evaluate the effectiveness of different classifiers with different input features, including one-hot encoded representations of GO/KEGG terms, functional embeddings, network embeddings, and the combination of network and functional embeddings. All the input features are first reordered using mRMR, resulting in the mRMR feature list. Then, a series of feature subsets are generated based on such list. On the series of feature subsets, several classifiers are built with a given classification algorithm. Each constructed classifier is assessed by 10-fold cross-validation. The measurements for each classifier, including accuracies on 16 categories, overall accuracy (ACC) and Matthew correlation coefficient (MCC) (Matthews, 1975; Gorodkin, 2004), are provided in Supplementary Tables 1–4. For each feature type and each classification algorithm, a curve is plotted with MCC as Y-axis and number of used features as X-axis, as shown in Figure 3. The MCC values change with the number of features for each classification algorithm. Clearly, for each feature type, RF outperforms other three classification algorithms.

**Figure 4:**
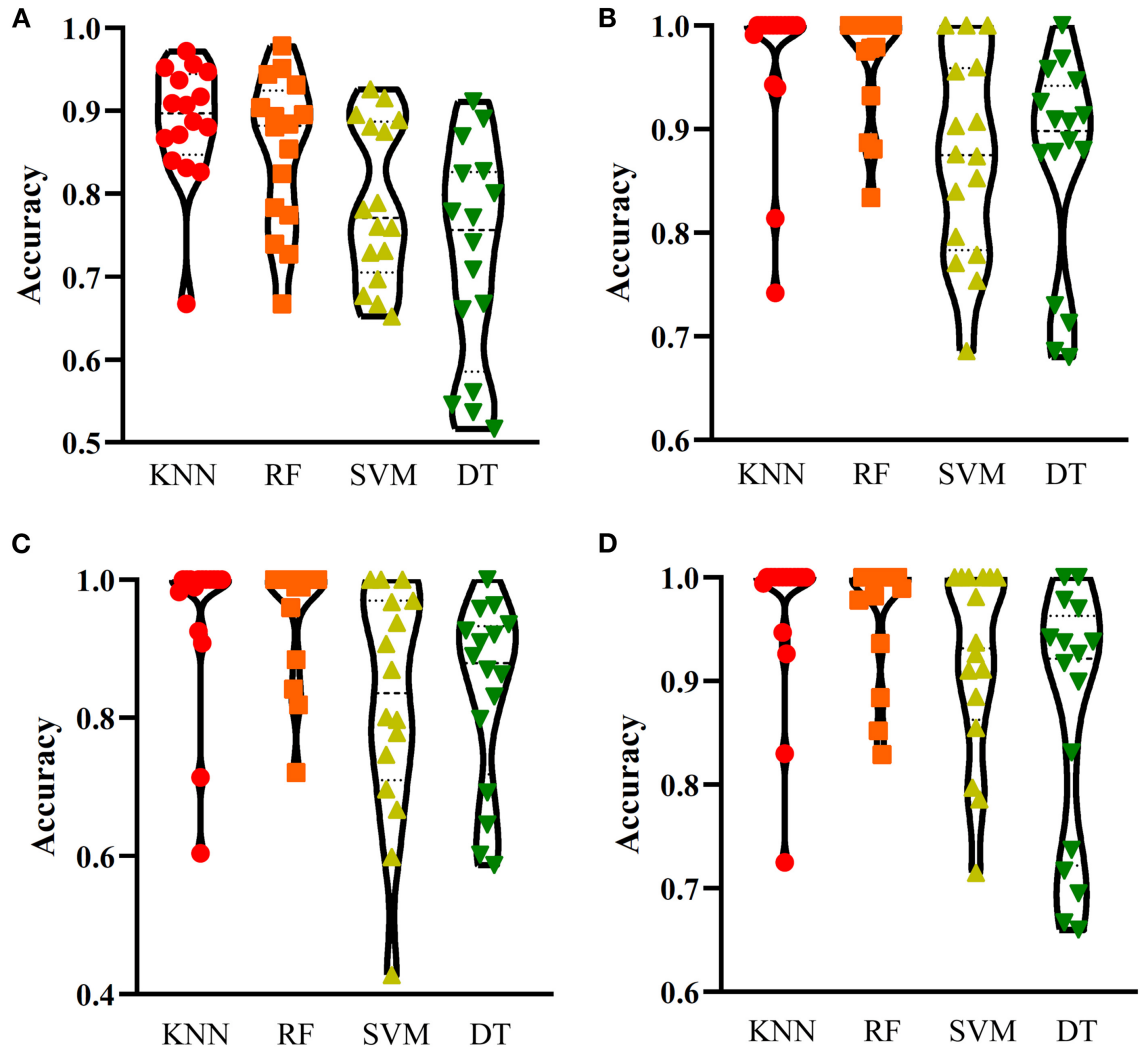
Performance of the optimum classifiers on 16 categories with different feature types. **(A)** one-hot encoded representation derived from KEGG/GO terms; **(B)** functional embeddings from KEGG/GO terms; **(C)** network embeddings from a protein-protein network; **(D)** the combined functional and network embeddings. KNN, K-nearest neighbors; RF, random forest; SVM, support vector machine; DT, decision tree.

For one-hot encoded representations of GO/KEGG terms, the corresponding curves are illustrated in Figure 3A. The optimum RF classifier yielded the MCC of 0.858, which uses the top 511 features. The corresponding ACC is 0.885 (Table 2). The MCCs of the optimum DT and SVM classifiers are 0.763 and 0.832, respectively, and the corresponding ACCs are 0.805 and 0.864. They are all lower than those of the optimum RF classifier. The MCC of the optimum KNN classifier is also 0.858, however, the ACC is only 0.882, lower than that of the optimum RF classifier. The accuracies of 16 categories yielded by four optimum classifiers are shown in Figure 4A, further confirming the superiority of RF.

**Table 2:** Comparisons of different classifiers with or without feature selection.

**Feature type**	**Classification algorithm**	**ACC**		**MCC**	
		**With feature selection**	**Without feature selection**	**With feature selection**	**Without feature selection**
One-hot encoded representations	Decision tree	0.805	0.776	0.763	0.726
	K-nearest neighbors	0.882	0.854	0.858	0.826
	Random forest	0.885	0.878	0.858	0.849
	Support vector machine	0.864	0.859	0.832	0.825
Functional embeddings	Decision tree	0.743	0.717	0.697	0.666
	K-nearest neighbors	0.860	0.852	0.837	0.828
	Random forest	0.897	0.889	0.876	0.867
	Support vector machine	0.799	0.798	0.762	0.760
Network embeddings	Decision tree	0.669	0.648	0.612	0.588
	K-nearest neighbors	0.786	0.785	0.755	0.754
	Random forest	0.835	0.827	0.803	0.795
	Support vector machine	0.669	0.661	0.618	0.609
Functional and network embeddings	Decision tree	0.746	0.720	0.699	0.670
	K-nearest neighbors	0.858	0.832	0.835	0.805
	Random forest	0.893	0.884	0.872	0.861
	Support vector machine	0.825	0.823	0.793	0.791

Of the functional embeddings, Figure 3B shows the curve for each classification algorithm. It can be observed that the four optimum classifiers yield the MCCs of 0.697, 0.837, 0.762, and 0.876, respectively. The corresponding ACCs are 0.743, 0.860, 0.799, and 0.897 (Table 2), respectively. Likewise, RF still yields the best performance. The detailed performance (accuracies on 16 categories) of four optimum classifiers is listed in Figure 4B. Again, the optimum RF classifier produces the most high accuracies, indicating the advantage of RF.

For the third feature type (network embeddings), we also plot four curves, one curve corresponds one classification algorithm, as shown in Figure 3C. The highest MCCs for four classification algorithms are 0.612, 0.755, 0.618, and 0.803, respectively. Corresponding ACCs are 0.669, 0.786, 0.669, and 0.835 (Table 2), respectively. Also, the optimum RF classifier yields the best performance. We further list the accuracies on all categories produced by four optimum classifiers in Figure 4C. Clearly, the optimum RF classifier is superior to other optimum classifiers.

As for the last feature type (the combination of network and functional embeddings), four curves are plotted in Figure 3D. The optimum RF classifier generates the MCC of 0.872 and ACC of 0.893 (Table 2). The optimum KNN classifier yields a high MCC of 0.835. However, the other two optimum classifiers produce much lower MCC (lower than 0.800). The ACCs shows the same results (see Table 2). Accuracies on all categories are shown in Figure 4D. Similarly, the optimum RF classifier provides the best performance.

As mentioned above, the optimum RF classifier is all best for four different feature types. The optimum RF classifier on functional embeddings derived from KEGG/GO terms yields the best MCC value (0.876). This classifier is based on the top 239 features. The optimum RF classifier with the combined embeddings only yields an MCC value of 0.872 and is a little worse than the RF with only functional embeddings. However, it uses only the top 129 features, which is nearly half of the number of features of the optimum RF classifier with only functional embeddings (239). Thus, in this study, we use the combined network and functional embeddings as the final input features.

**Figure 5:**
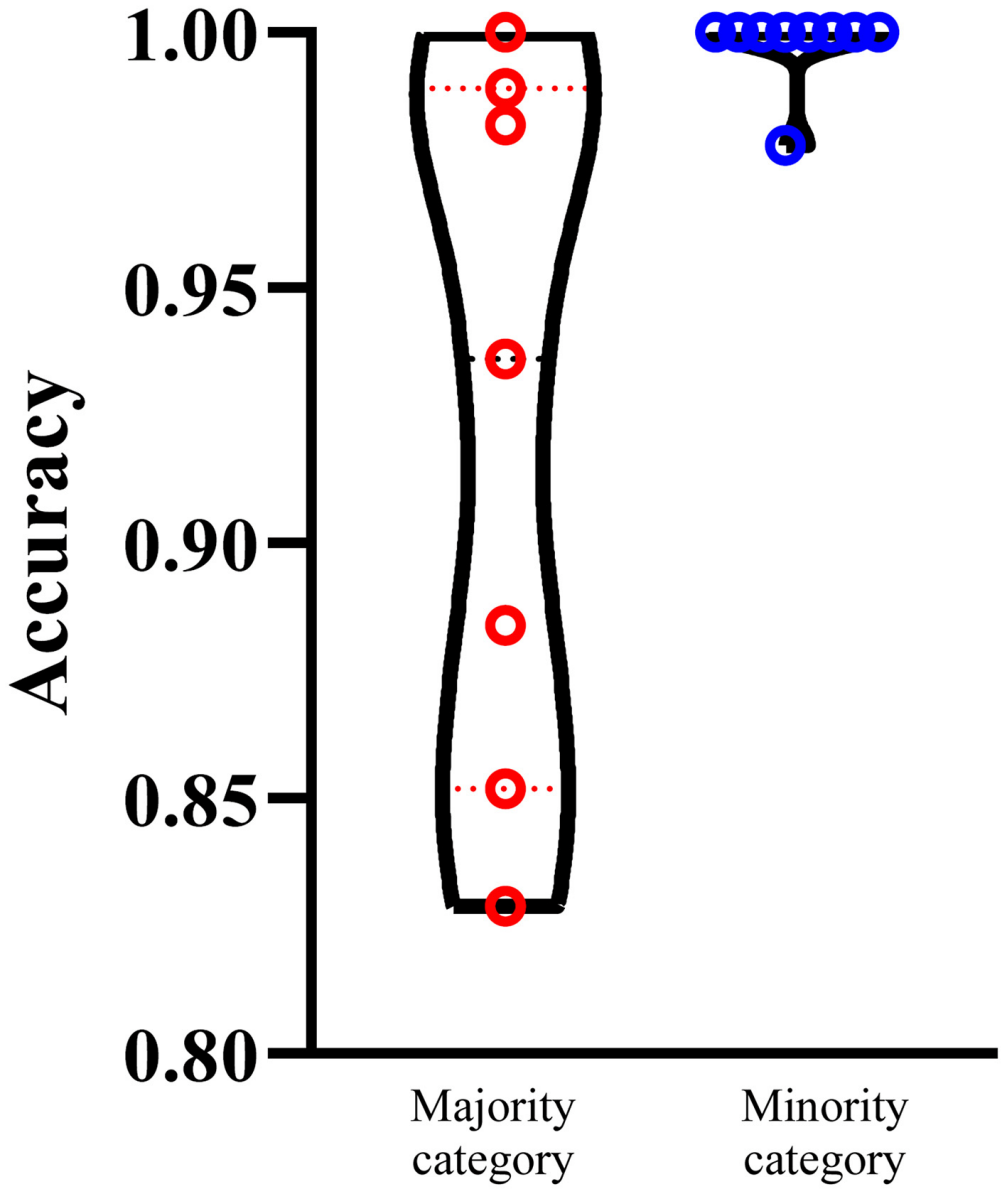
Performance of the optimum RF classifiers on majority and minority categories using the combined functional and network embeddings. The majority categories contain more than 100 proteins, whereas the minority categories consist of <100 proteins. The performance on minority categories is not lower than that on the majority categories.

We select the optimum RF classifier with the combined embeddings as the proposed method. As the sizes of 16 categories are of great difference, it is necessary to investigate the performance of such classifier on majority and minority categories. We set 100 as the threshold, that is, categories containing more than 100 proteins are deemed as majority categories, whereas other categories are termed as minority categories. In this case, we obtain seven majority categories and nine minority categories. The performance on majority and minority category of the proposed classifier is shown in Figure 5. It is surprising that the performance on minority categories is not lower than that on the majority categories. This result indicates that the performance of such classifier is not influenced by the imbalanced problem after SMOTE is applied.

### Comparison of Classifiers With or Without Feature Selection

In this study, we employed a feature selection procedure to improve the performance of different classification algorithms. Table 2 lists the performance of different classification algorithms on four feature types with or without feature selection. It can be observed that the performance of DT is enhanced most by the feature selection. MCC is improved about 3% and ACC is enhanced about 2.5%. The improvement on the performance of KNN yielded by feature selection is quite different for different feature types. For one-hot encoded representations and combined functional and network embeddings, the performance is evidently enhanced, while the performance is improved limited for other two feature types. As for other two classification algorithms (RF and SVM), the improvement is not very evident (almost all <1% for both ACC and MCC). Anyway, it can be confirmed that the employment of feature selection can improve the performance of all classification algorithms.

### Proposed Method Is Superior to State-of-the-Art Methods

To demonstrate the power of our proposed method, we compare our method with published methods, including BLAST and Deeploc. The results are listed in Table 3. BLAST and DeepLoc nearly have the same level of performance and are inferior to our proposed method. Of the 16 locations, our method can achieve 100% accuracy on nine locations. Here, DeepLoc has the worst performance, and a potential reason is that our benchmark dataset is heavily imbalanced and results in biased preference for majority classes. To resolve the data imbalance issue, our method applies SMOTE in the construction of a balanced training set.

**Table 3:** Performance of BLAST, DeepLoc, and our proposed method.

**Class**	**BLAST**	**DeepLoc**	**Ours**
Biological membrane	0.843	–	0.829
Cell periphery	0.424	–	1.000
Cytoplasm	0.455	–	0.852
Cytoplasmic vesicle	0.232	–	1.000
Endoplasmic reticulum	0.532	–	0.989
Endosome	0.280	–	1.000
Extracellular space or cell surface	0.739	–	0.936
Flagellum or cilium	0.000	–	1.000
Golgi apparatus	0.379	–	1.000
Microtubule cytoskeleton	0.333	–	1.000
Mitochondrion	0.356	–	0.982
Nuclear periphery	0.097	–	1.000
Nucleolus	0.241	–	1.000
Nucleus	0.733	–	0.884
Peroxisome	0.289	–	0.978
Vacuole	0.333	–	1.000
Overall accuracy	0.660	0.659	0.893
MCC	0.576	0.568	0.872

## Conclusion

In this study, we present an embedding-based method to predict protein subcellular locations by integrating protein interactions and functional information. The proposed method first learns network embeddings from a protein–protein network and functional embeddings from associations between proteins and GO/KEGG terms. We demonstrate that our proposed method is superior to state-of-the-art methods, and the learned embeddings offer valuable biological insights.

## Data Availability Statement

The datasets analyzed for this study can be found in the (Swiss-Prot) (http://cn.expasy.org/).

## Author Contributions

TH and Y-DC designed the study. XP, HL, and TZ performed the experiments. XP, HL, ZL, and LC analyzed the results. XP and HL wrote the manuscript. All authors contributed to the article and approved the submitted version.

## Conflict of Interest

The authors declare that the research was conducted in the absence of any commercial or financial relationships that could be construed as a potential conflict of interest.

## Supplementary Material

Supplementary Table 1 The performance of four classifiers change with the number of one-hot encoded representation derived from KEGG/GO terms.

Supplementary Table 2 The performance of four classifiers change with the number of functional embeddings from KEGG/GO terms.

Supplementary Table 3 The performance of four classifiers change with the number of network embeddings from a protein-protein network.

Supplementary Table 4 The performance of four classifiers change with the number of combined functional embeddings and network embeddings.
